# Enhancing COVID-19 tracking apps with human activity recognition using a deep convolutional neural network and HAR-images

**DOI:** 10.1007/s00521-021-05913-y

**Published:** 2021-03-30

**Authors:** Gianni D’Angelo, Francesco Palmieri

**Affiliations:** Department of Computer Science, University of Salerno, Fisciano, Salerno Italy

**Keywords:** COVID-19, Tracking app, CNN, Health monitoring system, Health monitoring devices, Healthcare, e-Health, IoT

## Abstract

With the emergence of COVID-19, mobile health applications have increasingly become crucial in contact tracing, information dissemination, and pandemic control in general. Apps warn users if they have been close to an infected person for sufficient time, and therefore potentially at risk. The distance measurement accuracy heavily affects the probability estimation of being infected. Most of these applications make use of the electromagnetic field produced by Bluetooth Low Energy technology to estimate the distance. Nevertheless, radio interference derived from numerous factors, such as crowding, obstacles, and user activity can lead to wrong distance estimation, and, in turn, to wrong decisions. Besides, most of the social distance-keeping criteria recognized worldwide plan to keep a different distance based on the activity of the person and on the surrounding environment. In this study, in order to enhance the performance of the COVID-19 tracking apps, a human activity classifier based on Convolutional Deep Neural Network is provided. In particular, the raw data coming from the accelerometer sensor of a smartphone are arranged to form an image including several channels (HAR-Image), which is used as fingerprints of the in-progress activity that can be used as an additional input by tracking applications. Experimental results, obtained by analyzing real data, have shown that the HAR-Images are effective features for human activity recognition. Indeed, the results on the k-fold cross-validation and obtained by using a real dataset achieved an accuracy very close to 100%.

## Introduction

**Fig. 1:**
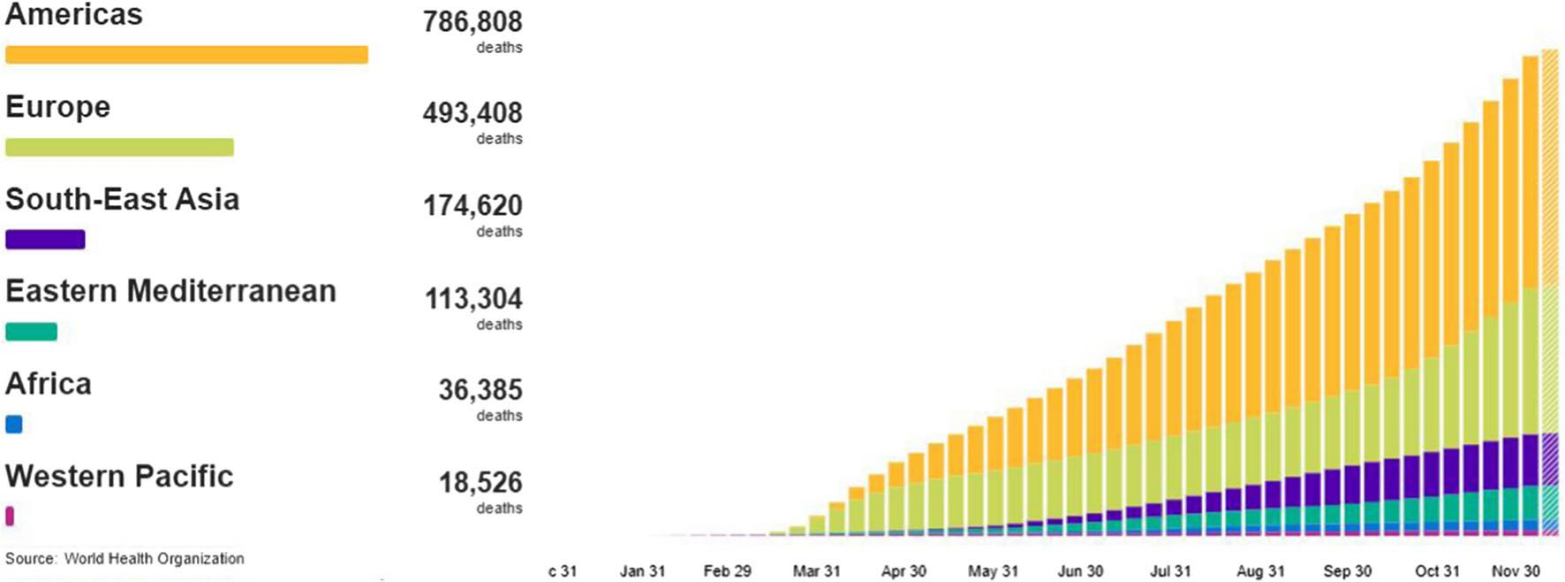
Cumulative number of deaths for Region. Source: World Health Organization

The COVID-19 outbreak has pushed health authorities to fight an unprecedented battle against the time. Since its first occurrence in Wuhan, on December 31, 2019, SARS-CoV-2 virus has spread in more than 200 countries around the world, with a case fatality rate (CFR) of 2.25% and an infection fatality rate (IFR) of 0.68% [38]. As reported by the World Health Organization (WHO), at the time of writing (December 16, 2020) there have been 71.919.725 confirmed cases of COVID-19 in the world, including 1.623.064 deaths [60]. As depicted in Fig. 1, the number of deaths is constantly increasing. Numerous countermeasures have been undertaken in the last months to cope with the virus pandemic, pending the long-awaited vaccine. Although the biological and medical fields are the more active ones [47], many other disciplines are involved in providing useful support to the issue [13, 21]. Under this aspect, the COVID-19 has changed how the world does science. Hundreds of clinical trials have been launched around the globe, gathering together thousands of researchers, medicals, hospitals, and laboratories. Never before, scientists from all over the world focused on a single topic and stopped almost all other researches.

Despite these efforts and even though some vaccines seem to be starting to spread around the world, the tracking of infected people seems yet the most affordable approach to take under control the spread of the pandemic. In this direction, solutions derived from modern mobile communications technologies seem to be the most promising options available [26, 41, 43, 53]. In particular, mobile health applications for smartphones have been adopted by various States around the world to provide useful tools for contact tracing, information dissemination, and pandemic control in general [18]. For example, in Singapore, a mobile app, called “TraceTogether,” is used to track the virus spreading after an individual was infected. “Immuni” is a similar app endorsed by the Italian government. In Switzerland, “SwissCovid” is the official mobile app managed by the Federal Office of Public Health (FOPH). Further, in the U.S. and UK, “COVID Symptom tracker” has been deployed by the Coronavirus Pandemic Epidemiology Consortium (COPE). A research on the most popular app stores, such as Apple AppStore and Google Play Store of the keywords Covid, SARS-CoV-2, and similars can provide an idea of the huge amount of apps available related to the COVID-19. The rapid proliferation of these apps has exacerbated the well-known problem related to app-quality assessment [11]. So that, in order to cope with it, a new metric named Mobile App Rating Scale (MARS) has been recently introduced [51]. A systematic review of the most popular apps for COVID-19 can be found in [18].

The main aim of these apps is to inform users if they have been close to an infected person for a time sufficient to be considered at high risk of contagion. Therefore, distance and position estimation are required to be as more accurate as possible. Although the positioning task has largely been solved for outdoor situations by using the Global Positioning System (GPS) along with precision refining methodologies [36], other technologies need to be used indoor. Typically, the indoor positioning systems (IPS) include a network of devices equipped with different technologies, which can be based on radio waves, such as WiFi and Bluetooth Low Energy (BLE) as well as on optical and acoustic solutions, like, for example the LiFi technology [40] and ultrasonic sensors, respectively. Nevertheless, as it is known, the distance between individuals can be estimated by using the Time-of-Flight (TOF) method without using the position of the involved devices. Due to its low energy consumption, easy deployment, low cost and widespread availability, BLE is the most widely used solution for distance estimation. More specifically, the RSSI (Receive Signal Strenght Indicator) is evaluated to estimate the distance between two devices/individuals through the Friis’s equation [24]. Nevertheless, the environment in which a BLE system operates has a decisive influence on the intensity of the received signal and, then, on the RSSI correctness. This can create problems when trying to estimate the distance. Some factors that can generate variability in the estimation of signal intensity are [50]:

- Metals and other reflective materials, which cause signal bounce;
- Liquid elements that absorb the signal. In this regard, people crowds represent a serious drawback. We remark that the human body, being composed mostly of water, constitutes an important barrier to the propagation of Bluetooth signals at 2.4 GHz - ISM Band;
- Physical obstacles.
- Height difference between devices.
- Relative orientation.

Although advances in the field of Machine Learning (ML), Deep Learning (DL), and Artificial Intelligence (AI) in general, have allowed the development of RSSI Real-Time Correction Algorithms [34], IPS and its applications to distance estimation remain a challenging problem.

Besides, most of the worldwide recognized social distance-keeping laws and criteria plan to keep a different distance based on the activity of the person and on the surrounding environment. For example, the distance between two individuals can be reduced if the facemask is used or if the persons are in outdoor spaces, whereas the distance must be increased if they are indoor or they are making physical activities, such as walking, running, footing, or other sports.

Therefore, information about the surrounding environment and user activity can heavily affect the utility of mobile health applications.

In this study, in order to enhance the performance of the COVID-19 tracking apps, a human activity classifier based on Deep Convolutional Neural Network (DCNN) is provided. In particular, the raw data coming from the accelerometer sensor of a smartphone are arranged to form a multi-channel image (HAR-image), which is used as a fingerprint of the in-progress activity. Our aim is to provide a system able to automatically learn useful information from the device’s owner concerning his activity and, then, also the surrounding environment. Such a system can be integrated as an additional function into tracking applications implementing the exposition estimation rules in order to empower their capabilities.

The comparison with the most common machine learning-based classifiers has shown that the aforementioned HAR-images are extremely effective features for Human Activity Recognition (HAR). Indeed, when evaluated through the k-fold cross-validation technique it achieved an accuracy very close to 100%.

The remainder of the paper is organized as follows. Firstly, in Sect. 2, the contact tracing is presented and discussed. In Sect. 3, the state-of-the-art methods for the HAR based on Machine Learning and Artificial Intelligence are shown. In Sect. 4, the HAR-Images and DCNN are presented in detail, whereas Sect. 5 describes the experiments and reports the results. Finally, Sect. 6 is devoted to the conclusions and future works.

## Contact tracing

Contact tracing is the main countermeasure adopted from public health to cope with COVID-19 disease [30]. More specifically, contact tracing refers to the ability to reconstruct the contact chains of virus-positive people. The tracking can also take place in a “traditional” way, by interviewing positive people and tracing the situations in which they could endanger the health of close people and properly warning them. Nevertheless, there are situations, such as having been in line at the supermarket, in a bar or office, in which we do not know exactly who we met. In these situations, digital support systems, such as an app, can represent an effective help. A contact tracing app works as follows: when a user downloads the app, an own code (id-code) is associated with it and such code varies several times a day. Using Bluetooth technology the smartphones that are in Bluetooth action range exchange these codes, without anyone, not even the system, knowing who they correspond to. To preserve privacy, the app does not collect any personal data, such as name, age, address, telephone number, or email. When a person is found to be positive to COVID-19, his id-code is entered by the healthcare staff into a system from which each smartphone picks up it and compares it with the list of codes it has registered. If it turns out that the smartphone has had risky contact with the one associated with a positive person, appropriate behavioral indications are provided to the involved user.

Nevertheless, there are two types of apps, that is apps that make use of centralized or decentralized databases. In both cases, when individual A meets B, their smartphones exchange encrypted id-codes [42]. If A becomes positive, the status of his app is updated. At this point, in centralized systems, the data remain on the server, in decentralized systems the data remain on the user’s smartphone.

**Fig. 2:**
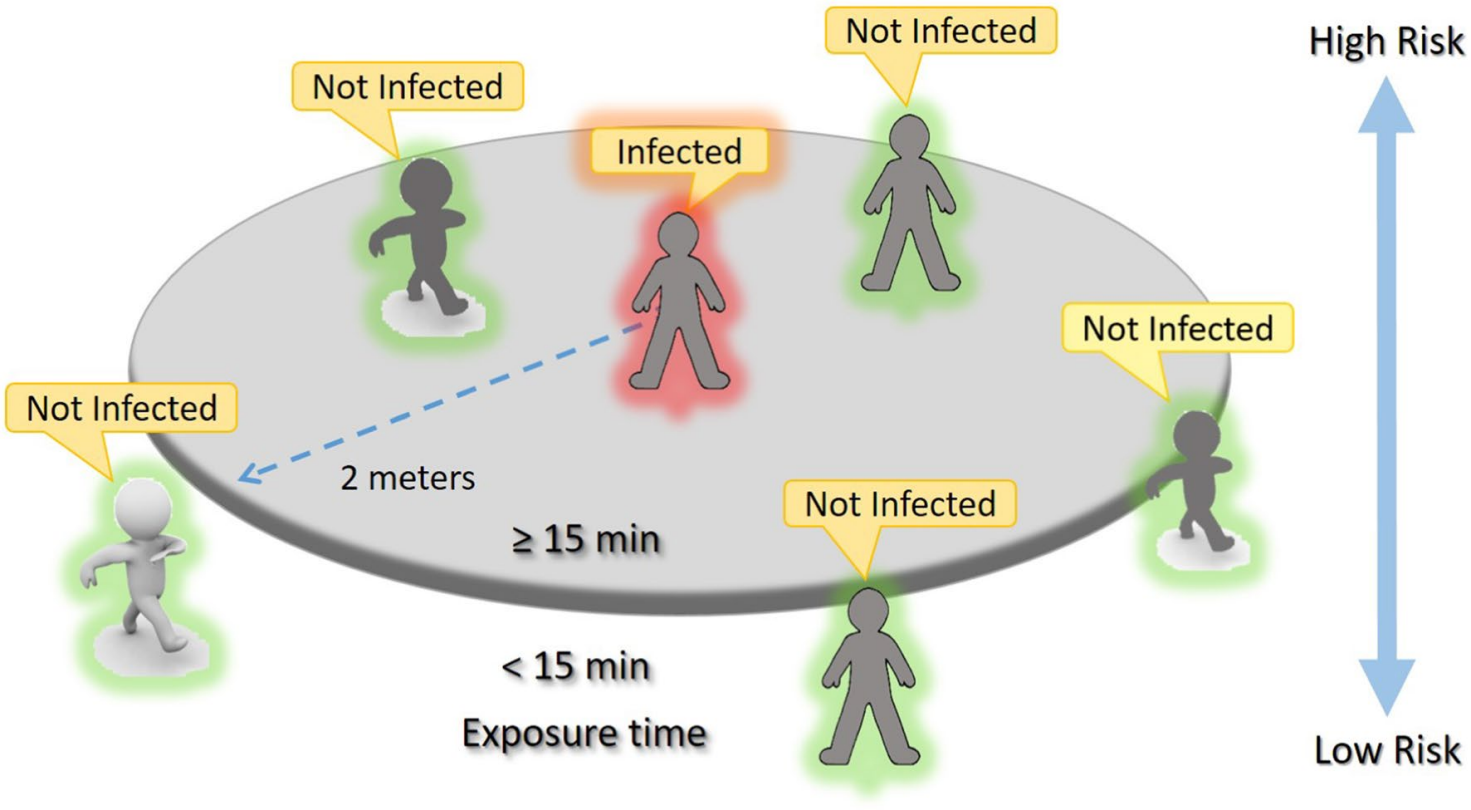
The ECDC Exposure risk indications. People that are being within a radius of 2 m from an infected person and for a time greater than 15 min are considered subjects with a high risk of contagion

Regardless of the type, the apps need to respond to the guidelines emitted from worldwide health organizations. For example, with reference to Fig. 2, the European Centre for Disease Prevention and Control (ECDC) indicates the following criteria for establishing if COVID-19 exposure is to be considered to have a high-risk level:

- A person living in the same family as a COVID-19 case;
- A person who has had direct physical contact with a COVID-19 case (for example, shaking hands).
- A person who has had unprotected direct contact with the infectious secretions of a COVID-19 case (for example, who coughed, touched used paper handkerchiefs with bare hands);
- A person who has had face-to-face contact with a COVID-19 case within 2 m and for more than 15 min.
- A person who was in an enclosed environment (e.g., classroom, meeting room, hospital waiting room, etc.) with a COVID-19 case for 15 min or more and at a distance of fewer than 2 m.
- A healthcare worker or other person providing direct assistance to a COVID-19 case, or laboratory workers handling COVID-19 case samples without the recommended Personal Protective Equipment (PPE) or with a possible infringement of PPE;
- Contact in an aircraft occurred in two contiguous seats (in any direction) from a COVID-19 case, travel companions or assistants and crew members on duty in the section of the aircraft where the case in question occurred (if the severity of symptoms or the movement of the case indicates more extensive exposure, the passengers seated in the entire section or all passengers on the aircraft can be considered in close contact).

On the other end, the cases considered to be at low-risk are:

- A person who has been in an enclosed environment with a COVID-19 case for fewer than 15 min or at a distance greater than 2 m.
- A person who has had face-to-face contact with a COVID-19 case for fewer than 15 min and at a distance of fewer than 2 m.
- Traveling together with a COVID-19 case in any type of transport over a distance of greater than 2 m.

Finally, the ECDC explains that a “longer contact duration increases the risk of transmission.” The 15-min limit is arbitrarily chosen for practical purposes.

Notice that, according to World Health Organization, the incubation period, during which a person is however contagious, ranges from 2 to 14 days. It is not easy to know and remember who we have been close to, within 2 m, for about 15 min, in the last two weeks. For this reason, an app able to estimate the proximity of two devices can give an effective contribution. Further, a point-to-point detection system eliminates the need for central data collections by States and, also, it can be sufficient for tracing people by considering the threshold of 15 min of exposure. Indeed, the goal of these applications is not to warn people that certain distance thresholds have been exceeded, but to be able a posteriori, many days later, to warn that a proximity contact has occurred with people who are being tested positive in a time later the contact.

## Human activity recognition and related work

In estimating exposure risks, an effective contact tracing cannot prescind from the considerations concerning the activities that characterize the specific behavior of the involved users (if they are running, or walking, standing or sitting, etc.). Thus, automatic HAR assumes paramount importance in supporting tracking applications.

Although the human activity recognition task has been extensively studied in the last decades [28], it remains a challenging problem that needs to be addressed and solved. Its main use is in eldercare and healthcare applications, especially when it is combined with other technologies, such as the Internet of Things (IoT). HAR can be performed by using many technologies, but nowadays the proliferation of small sized electronic equipment and the large usage of AI- and ML-based algorithms in many research and industrial fields [12, 14–16], have allowed the spread of HAR solutions by leveraging the built-in sensors of smartphones. Typical human activities that can be recognized by HAR systems are: walking, sleeping, driving, sitting, running, standing, cooking, etc. Nevertheless, HAR is widely used in many other real applications, such as crime monitoring [39], domotic [22], suspicious human activity [55], daily activities [17], gesture recognition [31], and military applications [35].

Data collected by inertial systems, video frames, and images are usually processed to make HAR. In this regard, HAR is generally performed firstly by processing univariate or multivariate time series in order to extract useful and effective features, and, then, by making activities inference through ML-based techniques. Deep neural networks (DNN) are the most promising tools for automatic features learning starting from raw data, and they have outperformed the signal processing approach, which requires a great knowledge of specific domains and a manually designing of the features [58].

The current approaches to make HAR can be classified into two main categories, that is, *vision-based* and *sensor-based*. The core processing of the former includes the classical steps adopted in computer vision for mining useful features, such as data preprocessing, cleaning, segmentation, feature extraction, and finally classification. Due to the huge diffusion of the built-in cameras in mobile electronic devices, in the last decades, there has been a great demand for automatic image processing, and then many approaches have also been proposed for video-based HAR technologies. The most significant advances in this field can be found in [5], where the HAR task is categorized according to several criteria. A taxonomy-based approach and an enlightening comparison among different methods are provided by Aggarwal, et. al. in [1]. Again, in [29], the authors discuss the advantages and the disadvantages of different features mining from images. Many other surveys have been found in the literature [7, 48, 52, 56]. However, as reported in [57], issues related to shadows, observing angle, background colors, light intensity, and more can negatively affect the HAR quality.

In contrast, built-in sensors in smartphones can overcome these issues and can be effectively used for HAR. Besides, many daily activities, such as sitting, standing, walking are strongly related to gravity and accelerations, and then they can be identified by using three-axis accelerometers, gyroscopes, and other sensors commonly integrated into smartphones. A detailed description of how the accelerometers of smartphones are used for HAR can be found in [4]. While in [37], the combined usage of accelerometer, gyroscope, and magnetometer sensors along with a deep neural network is shown. Also, in [3], the authors propose a HAR system capable to identify 20 activities by using five wearable dual-axis accelerometers and a ML-based classifier, achieving an accuracy of 84%. Again, in [49], five transport activities are identified by only using the smartphone inertial sensors.

Another important aspect to be considered in sensor-based HAR is that the time series-based classification requires the partitioning of the signals in temporal windows, which are associated with an activity. Usually, fixed-length temporal windows, that are shifted on time series, are used (sliding window approach). For example, in [25] a HAR system able to recognize six activities applying a Deep Recurrent Neural Network (DRNN) is presented. The paper shows how the size of the sliding window and its offset (so-called *stride*) affect the recognition time (throughput). The best recognition rate of 95.03% was achieved. In [8], the time series of each axis of a three-axis accelerometer were partitioned by a 50-dimensional window, corresponding to 2.5 seconds, and, then, given as input to three different recurrent networks, one for each axis. The model has proven to be able to distinguish six activities with an accuracy of 95.1%. Nevertheless, a dynamic sliding window is shown in [44]. The approach proposed by the authors is able to adjust the window size and the stride at every step of the training. The experimental results have shown that the model is able to achieve good performance. Indeed, the best result was 95.32% and 97.69% for the recall and precision metrics, respectively.

However, as reported in [2], the choice of the right window size and offset is an open challenge, because it depends on many factors, such as the nature of the sensor data, the activity to be recognized, and the designed model.

Finally, for their similarity to the approach shown in this study, we report some of the most notable methods using Convolutional Neural Networks (CNNs) in HAR. Although CNN has been originally designed for dealing with images, its use in many other application fields has proven to be very effective. Indeed, 1D signals can also be processed by a CNN by exploiting the aforementioned sliding window approach. The first important study reporting the usage of CNN for HAR can be found in [63]. The authors use different CNNs for each axis of the accelerometer, and then the CNN outputs are arranged in a unique flattened vector, which is used as a hidden layer of a fully connected neural network. The achieved accuracy is 88.19%, 76.83%, and 96.88% for three different datasets, respectively. The divide and conquer approach together with a 1D CNN is used in [9]. The activities are first classified into static and dynamic classes by a binary 1D CNN-based classifier, and then for each class, specific activities are discriminated by a 3-class 1D CNN classifier. The results show an accuracy of 97.62% and 94.2% when tested on UCI and OPPORTUNITY datasets, respectively. Images derived by applying the Fast Fourier Transform (FFT) to each signal of the three-axis accelerometer and gyroscope are used in [27]. The resulting 28 x 28-dimensional images are used as input to the CNN to achieve performance values of 88%, 87%, and 87% for precision, recall, and F1 score, respectively, when the method is applied for recognizing eight human activities.

A comprehensive study of different techniques used for making HAR based on machine learning algorithms and deep networks can be found in [54].

## HAR-images based activity recognition

In this Section, the proposed approach is shown in detail. To this end, the mathematical representation of HAR is first provided, next the HAR-Images are described, and finally how they are used in CNN in order to mine effective features is explained.

### Mathematical formalism of HAR

According to the definition given in [10], human activities can be seen as a set of actions performed by the user in a given environment over a temporal period.

Accordingly, let $A=\{A_1,A_2,\dots ,A_n\}$ be the set of the activities to be recognized, and let $S=\{S_1,S_2,\dots ,S_m\}$ be the set of the sensors involved in data capturing related to the activities.

Then, a specific action, $a \in A$, that occurs in a certain time window ($\varDelta t$) can be associated to a tuple of time-series ($d^{\varDelta t}$) capturing the activity information, whose elements ($r_l^t$) are the sensor readings acquired over $\varDelta t$.

1 $$\begin{aligned} d^{\varDelta t}=<r_i^t,r_j^t,\dots ,r_k^t>, with \; i, j, k \; \in S \; and \; t \in \varDelta t \end{aligned}$$

Note that because sensors can provide multiple time series as a result of the measurement action, then any $r_l^t$ is a $q^{(l)}$-dimensional vector of time series, with $q^{(l)}$ the number of time series provided from the sensor $l \in S$. Besides, because any sensor has a proper operating sample frequency ($f^{(l)}$), the number of sample ($\nu ^{(l)}$) captured in the given temporal window $\varDelta t$ is given by:

2 $$\begin{aligned} \nu ^{(l)}=\varDelta t * f^{(l)} \end{aligned}$$

**Fig. 3:**
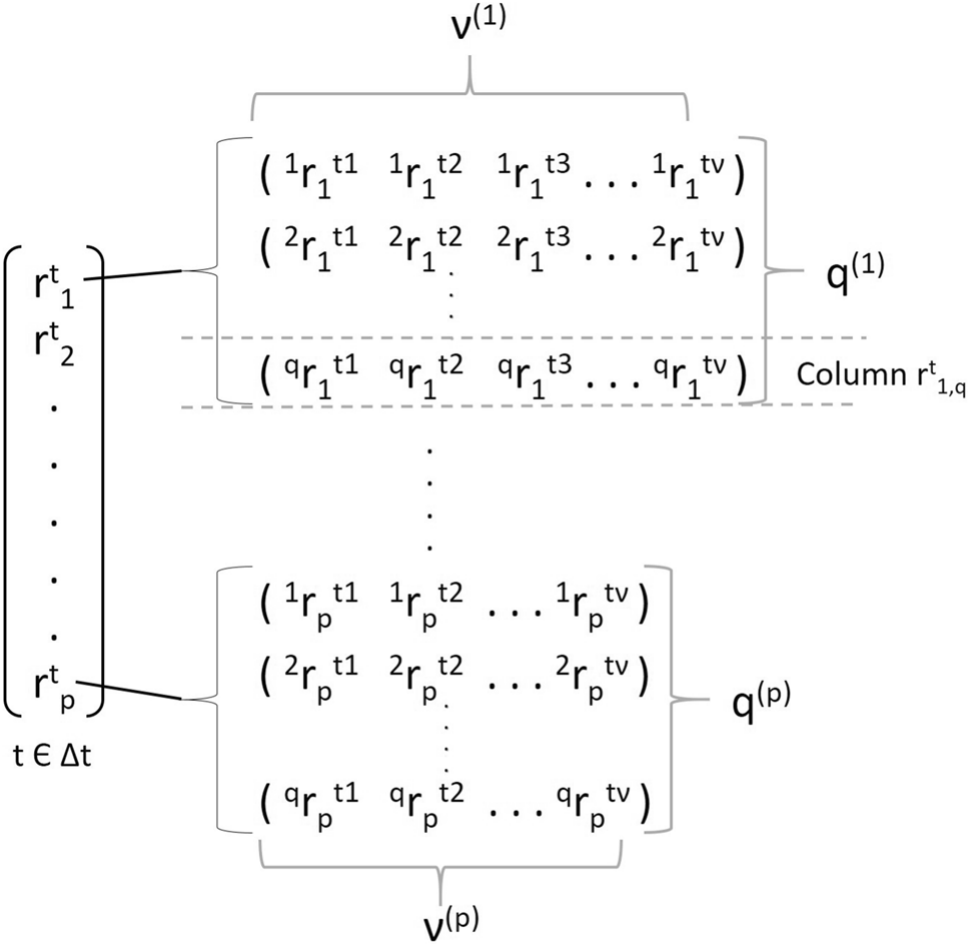
Data structure of $d^{\varDelta t}$. There are *p* sensors involved. Any sensor reading ($r_l^t$) is associated with a different number ($q^{(l)}$) of time-series of length equal to $\nu ^{(l)}$

As a consequence, any $r_l^t$ is a $(\nu ^{(l)} \times q^{(l)})$-dimensional matrix, while $d^{\varDelta t}$ can assume the aspect of a $(\nu \times q)$-dimensional matrix, where $\nu =Max(\nu ^{(l)}),\; \forall l \in S$, and $q=\sum _{l=1}^{p}q^{(l)}$, with *p* the number of sensors involved in the measurement. More specifically, $d^{\varDelta t}$ can be represented by a tensor having the structure as depicted in Fig. 3.

The goal is to build a map ($\varSigma$) between the action occurred over $\varDelta t$, $\tilde{a}^{\varDelta t} \in A$ and the sensor readings, $\tilde{d}^{\varDelta t}$, that is:

3 $$\begin{aligned} \tilde{a}^{\varDelta t}=\varSigma (\tilde{d}^{\varDelta t}) \end{aligned}$$

Note that $\varSigma$ provides an estimation of the action associated with the sensor reading. So that, $\tilde{a}^{\varDelta t}$ is to be considered as the estimated action, which needs to be compared with the corresponding true action $\widehat{a}^{\varDelta t}$ (ground truth).

Hence, the HAR goal is to find out an optimal $\varSigma$ by minimizing the discrepancy between the estimated action ($\tilde{a}^{\varDelta t}$) and the true one ( $\widehat{a}^{\varDelta t}$).

Typically, a loss function ($\Gamma$) is employed for representing this discrepancy, as follows:

4 $$\begin{aligned} loss \; function = \Gamma (\varSigma (\tilde{d}^{\varDelta t}),\widehat{a}^{\varDelta t}) \end{aligned}$$

Besides, the time series are not directly used as input to $\varSigma$, but a their projection ($\varPhi$) on a new *d*-dimensional state space is employed. That is:

5 $$\begin{aligned} loss \; function = \Gamma (\varSigma (\varPhi (\tilde{d}^{\varDelta t})),\widehat{a}^{\varDelta t}) \end{aligned}$$

with

6 $$\begin{aligned} \varPhi (\tilde{d}^{\varDelta t})=\{\varphi _i\}_{i=1}^d \end{aligned}$$

where $\varphi _i$ are the new features.

Ultimately, the goal is to minimize the equation (5) for any $a \in A$.

### HAR-images

Usually, sensor-based HAR is considered as a classical time series analysis problem, so that it is addressed by using recurrent neural networks, such as LSTM (Long Short-Term Memory) [8, 25, 62]. On the contrary, in this study, we investigate the feasibility of recognizing human activities by using convolutional neural networks. To this purpose, images need to be derived from sensor raw data. We referred to them as HAR-Images.

With reference to equation (1), any sensor reading is first windowed and then used to build the HAR-Images as follows. As mentioned earlier, any $r_l^t$ is a $(\nu ^{(l)} \times q^{(l)})$-dimensional matrix, so that two generic columns, $r_{l,j}^t$ and $r_{l,k}^t$, of this matrix, may be used to build an image on an x-y plane. The dots on this plane are depicted by using a column as the x-coordinate and the other one as the y-coordinate. Hence, for any temporal window ($\varDelta t$), the derived image includes $\nu ^{(l)}$ dots. Besides, for a sensor $l \in S$ with $q^{(l)}$ readings, the number of distinct admissible column couples, and then of images ($nC^{(l)}$) is given by:

7 $$\begin{aligned} nC^{(l)}=\frac{q^{(l)}(q^{(l)}-1)}{2} \end{aligned}$$

Note that the image size of each channel depends on the minimum and maximum values assumed by each column involved in the given temporal window ($\varDelta t$). Indeed, we remark that the coordinates of any dot are individuated by the values of these columns at a given index. Therefore, let $\eta ^{(l)}$ be the difference between the maximum and the minimum values assumed by the reading values of a sensor *l*, then the size of each corresponding image-channel ($chSize^{(l)}$) is given by:

8 $$\begin{aligned} chSize^{(l)}=\eta ^{(l)} \times \eta ^{(l)} \end{aligned}$$

However, in order to provide more informative content to image-channels, any dot on the x-y plane is associated with a number ranging from 0 to 255, as it happens in the matrix representation of images. Such a number represents the number of occurrences that a dot falls in the same position. However, because the sensor readings include different values, it could happen that no dots fall in the same position. To address this issue a quantization process is performed on the images. More precisely, each column is first quantized through a given quantization step (*Q*), and then the resulting discrete sets are used as coordinates of the dots. As a consequence, the size of the quantized image-channel ($chSizeQ^{(l)}$) becomes:

9 $$\begin{aligned} chSizeQ^{(l)}=\mu ^{(l)} \times \mu ^{(l)} \end{aligned}$$

with $$\mu ^{(l)}$$:

10 $$\begin{aligned} \mu ^{(l)}=\frac{\eta ^{(l)}}{Q} \end{aligned}$$

Ultimately, each image built on a couple of columns can be considered as a different channel of a HAR-image. The same procedure is adopted for each sensor. So that, for a given $\varDelta t$, the number of HAR-Images is *p*, and each HAR-Image includes $nC^{(l)}$ channels.

For example, for a three-axis accelerometer ($q=3$ and $p=1$), the different time series associated with each axis captured in a temporal window $\varDelta t$ lead to $nC=3$, that is a unique ($p=1$) HAR-Image including three channels. The first image-channel is derived by considering the axes X-Y, the second X-Z, and the third Y-Z.

With reference to equation (6), the HAR-Images represent a kind of projection ($\varPhi$) of the time series into a $(p \times \mu \times \mu \times nC)$-dimensional state space. Nevertheless, as it is shown in the next subsection, such a projection includes other transformations.

### HAR-features mining and deep convolutional neural network

Once the HAR-Images are built, relevant features need to be mining from them in order to build a classifier. Pattern recognition (PR) based approaches have been extensively used in the last decades for making HAR [33]. Although PR has proven to be effective in cases in which only a few activities need to be recognized, it has shown several weaknesses in most daily cases. Indeed, firstly, the features are mining through a heuristic hand-crafted way. The effectiveness of this approach heavily can be affected by human skills in the specific domains. Secondly, often, such features are related to some statistical measure, such as standard deviation, mean which are not able to capture deep insight. Thirdly, traditional PR requires many labeled data, which could be not always available. Finally, most of the existing PR-based approaches make use of static data. As a consequence, they are not adequate for dealing with data streams and incremental learning.

On the contrary, deep learning is able to overcome these issues by making simultaneously the features extraction and the model building in a unique process [6]. Further, features are learned automatically, they can represent complex activities, and finally, the insight learned can be transferred to new similar tasks.

Among many deep learning-based approaches, we focus on the Deep Convolutional Neural Network, which is a stacked network including a Convolutional Neural Network and a Deep Neural Network. DNNs can be seen as a special category of the more general Artificial Neural Network (ANN). The main difference between them is that DNN makes use of more hidden layers than ANN. This confers to DNN the capability of mining more representative and salient features through a process in which more complex features are derived from less complex ones. Layers near to the input are representative of simple features, while layers closer and closer to the output represent more complex features. Generally, DNNs are used after other deep networks, such as CNNs.

CNNs are extensively used in computer vision, and they have proven high ability to make image classification, speech and text analysis. As it is known, CNNs rely on the convolutional operation followed by a pooling process.

As described earlier, for a sensor $l \in S$, the corresponding HAR-Image includes $nC^{(l)}$ image-channels (see equation (7)), each of size $(\mu ^{(l)} \times \mu ^{(l)})$. Thus, let $X^{c,(l)} \in {\mathcal {R}}^{\mu ^{(l)} \times \mu ^{(l)}}$ be the *c*-th single channel-image of the HAR-Image associated to the sensor *l*, and $K^{f,(l)} \in {\mathcal {R}}^{a\times b}$ be the *f*-th convolutional filter, then the convolution operation between the CNN-input ($X^{c,(l)}$) and $N_f$ filters produces the following output ($Y^{c,(l)}$):

11 $$\begin{aligned} Y_{i,j}^{c,(l)}=\sum _{f=1}^{N_f} \sum _{p=1}^a \sum _{q=1}^b K^{f,(l)}_{p,q} X_{i+p-1,j+q-1}^{c,(l)} \end{aligned}$$

with $Y_{i,j}^{c,(l)}$ the entries of the output matrix $Y^{c,(l)}$.

The row size ($Y_x^{c,(l)}$) and column size ($Y_y^{c,(l)}$) of $Y^{c,(l)}$ are given by:

12 $$\begin{aligned} \begin{aligned} Y_x^{c,(l)}=&\frac{\mu ^{(l)}-a+2P}{S_x}+1\\ Y_y^{c,(l)}=&\frac{\mu ^{(l)}-b+2P}{S_y}+1 \end{aligned} \end{aligned}$$

where $S_x$ and $S_y$ control the shifting (called *stride*) of the filter on both the x and y axes of the input. While, *P* (*Padding*) defines the number of zeros to add around the border of $X^{c,(l)}$ in order to match the output size to that of the input, without compromising the result of the convolution operation.

To take into account the presence of multiple channels of the sensor involved, the multi-channel 2D convolutional process is used. It consists of the element-wise summation ($Y^{(l)}$) of the output of each convolutional operation performed on each channel of the given sensor $l \in S$.

13 $$\begin{aligned} \begin{aligned}&Y_{i,j}^{(l)}=\sum _{c=1}^{nC^{(l)}} Y_{i,j}^{c,(l)}, \\&with \; i=1,\dots ,Y_x^{c,(l)} \; and \; j=1,\dots ,Y_y^{c,(l)} \end{aligned} \end{aligned}$$

where $Y_{i,j}^{(l)}$ the entries of $Y^{(l)}$.

Note that all image-channels of a HAR-Image associated with a sensor *l* have the same size. Thus, with an abuse of notation, we can stat that the size of $Y^{(l)}$ can be expressed as follows:

14 $$\begin{aligned} \begin{aligned} Y_x^{(l)}\equiv&Y_x^{c,(l)} \\ Y_y^{(l)}\equiv&Y_y^{c,(l)} \end{aligned} \end{aligned}$$

for any $$c=1,\dots ,nC{(l)}$$.

Once the convolutional operation is ended, the pooling process is generally performed. It is used to reduce the size of the convolutional output in order to speed up the computational tasks. To accomplish this, the CNN-output ( $Y^{(l)}$) is divided into regions on which some calculations are performed. A typical pooling is the *MaxPool*, which consists of finding out the maximum value in the considered region and using it as a point on the new image-output.

Ultimately, a pooling operation that uses a $(k^{(l)} \times k^{(l)})$-dimensional regions, reduces the $(Y_x^{(l)} \times Y_y^{(l)})$-dimensional CNN-output ($Y^{(l)}$) to $(Y_x^{(l)}/k^{(l)} \times Y_y^{(l)}/k^{(l})$-dimensional MaxPool-output $(W^{(l)})$, which represents the so-called *feature map*.

15 $$\begin{aligned}{}[W^{(l)}]\equiv [Y_x^{(l)}/k^{(l)} \times Y_y^{(l)}/k^{(l)}] \end{aligned}$$

Thus, with reference to equation (6), further transformations have been added to the raw sensor data. In a nutshell, $\varPhi$ acts in tho steps: a) the raw sensor data have been first arranged as images, b) and then such images are used to mine useful features. Note that what has been said so far refers to a single sensor $l \in S$.

To take into account all involved sensors, any feature map ($W^{(l)}$) associated with a sensor *l* is flattened in a one-dimensional vector. Then, all these vectors are joined together to form a new unique one-dimensional vector ($Flat\_Vec$). With reference to equation (15), the size of this vector can be expressed by:

16 $$\begin{aligned}{}[Flat\_Vec]= \sum _{l=1}^p (Y_x^{(l)}/k^{(l)} * Y_y^{(l)}/k^{(l)}) \end{aligned}$$

with *p* the number of sensors involved in the measure.

Finally, a DNN with a softmax output layer is connected to the CNN-output for classification purposes. More precisely, $Flat\_Vec$ is given in input to a deep fully connected neural network, and then its output is provided as input to the softmax network to infer the class of the input in terms of probabilities.

Accordingly, let $z=1,\dots ,\psi$ be the index of the hidden layers of a DNN, let $s^{(z)}$ be the number of neurons of each hidden layer (*z*), then when $Flat\_Vec$ is provided as input to this DNN, the output of each layer ($h^{(z)}$) can be expressed by:

17 $$\begin{aligned} h^{(z)}=\sigma (W^{(z)} \cdot h^{(z-1)} + b^{(z)}) \end{aligned}$$

where $W^{(z)} \in R^{s^{(z)} \times s^{(z-1)}}$ and $b^{(z)} \in R^{s^{(z)}}$ are the weights matrix and the bias vector of the layer *z*, respectively. While, $\sigma$ is an activation function. We remark that the size of $h^{(z)}$ is $s^{(z)}$. Besides, the layer $z=1$ takes as input the above $Flat\_Vec$.

As above-mentioned, the last layer ($\psi$) includes a softmax function. So that, with reference to equation (17), the *j*-th output component ($h_j^{(\psi )}$) of the last layer of the DNN can be expressed as follows:

18 $$\begin{aligned} h_j^{(\psi )}=\sigma (\sum _{k=1}^{|h^{(\psi -1)}|} w_{j,k}^{(\psi )} h_k^{(\psi -1)}+b_j^{(\psi )}) \end{aligned}$$

with $w_{j,k}^{(\psi )}$ and $b_j^{(\psi )}$ the entries of $W^{(\psi )}$ and $b^{(\psi )}$, respectively.

Then, the *j*-th component of the softmax output ($h^{sm}$) is given by:

19 $$\begin{aligned} h_j^{(sm)}\equiv A_j=\frac{e^{h_j^{(\psi )}}}{\sum _{k=1}^n e^{h_k^{(\psi )}}} \end{aligned}$$

with $j=1,\dots ,n$ and *n* the number of admissible actions (*A*).

The action ($A_j$) with higher probability is considered to be the output of the classifier. Such an action along with the true action are fed as input to the loss function of equation (5) and used during the training phase.

**Fig. 4:**
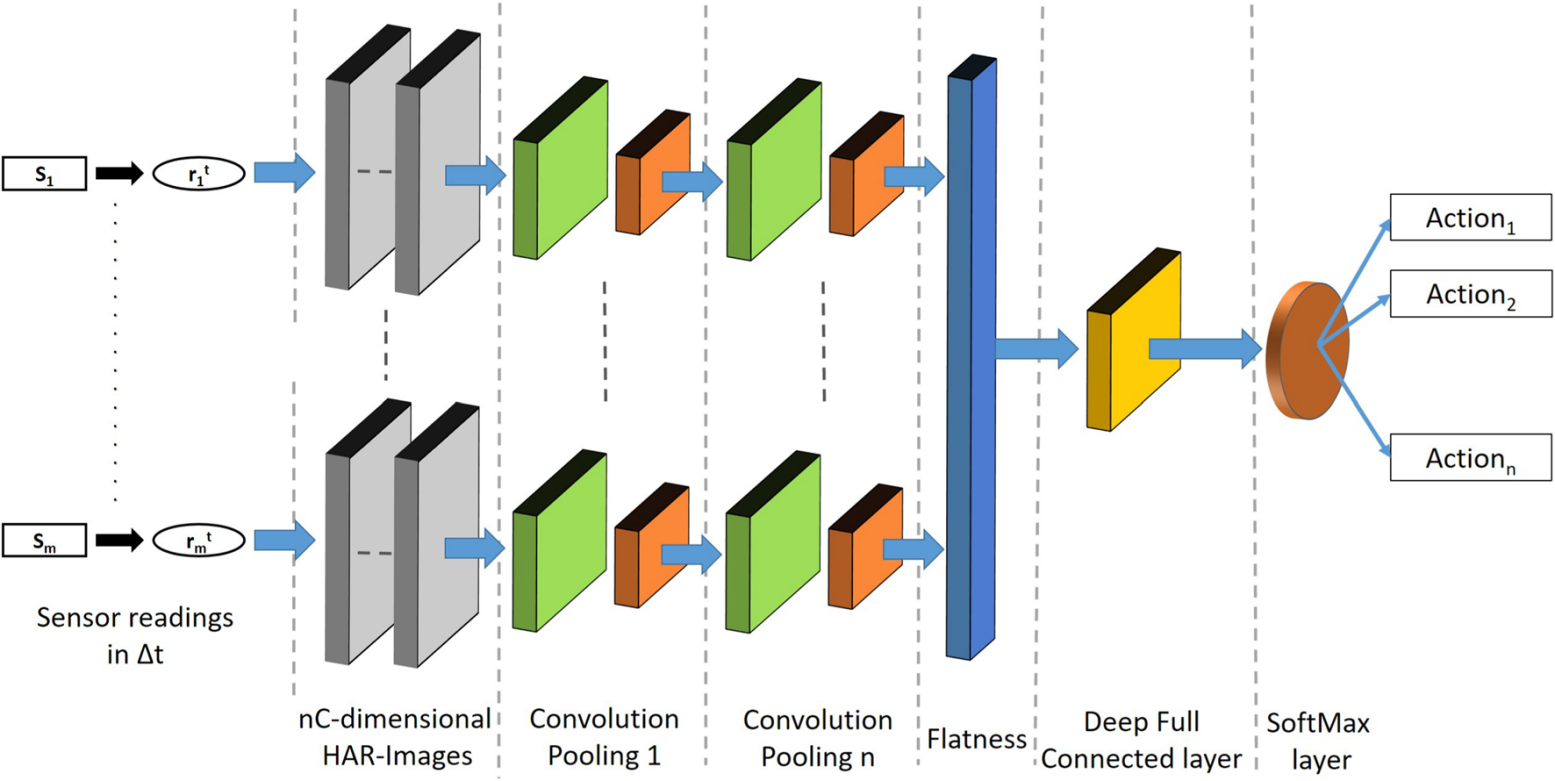
Network structure of HAR-Images DCNN. As depicted, once the HAR-images over a given $\varDelta t$ are built, several convolutional and pooling transformations are performed. The output of these layers is first flattened and then fed to DNN. The softmax layer concludes the network by providing an estimation of the classes in terms of probabilities

Figure 4 depicts the general structure of the DCNN used in this study. Note that the depicted network is used for any $\varDelta t$. That is to say that the network is trained through all the HAR-Images derived by sliding the temporal window $\varDelta t$ on all training dataset. To accomplish this, usually, a specific offset ($stride^{(l)}$) is used. As a consequence the number ($numHAR_{Images}^{(l)}$) of nC-dimensional HAR-Images associated with a sensor reading *l* is given by:

20 $$\begin{aligned} numHAR_{Images}^{(l)}=\left\lceil \frac{(D^{(l)}-\varDelta t)}{stride^{(l)}} + 1\right\rceil \ \end{aligned}$$

where $D^{(l)}$ is the length of the sensor reading *l*. As it is better shown in the next section, the choice of $\varDelta t$ and *stride*, and consequently of $numHAR_{Images}^{(l)}$, heavily affects the performance of the proposed network, and then they need to be chosen appropriately.

Finally, we remark that with reference to the equation (5), the loss function to be minimized is that generally used for training deep neural networks for multi-class classification [64].

## Experiments and results

The aim of this Section is to demonstrate the effectiveness of HAR-Images in representing actions starting from raw sensor readings and to show the effectiveness of the DCNN as a powerful classifier for HAR. To accomplish this, a real dataset was used in the experiments. All the experiments were conducted by using the high-level neural networks API library for Python over the TensorFlow platform running on a 64-bit Windows 10 operating system. The machine used to perform the experiments was a portable PC equipped with an Intel(R) 4-Core i7-8565U CPU @ 1.80GHz, 16 GB of RAM, and NVIDIA GeForce MX150 GPU with 4 GB of memory.

### WISDM dataset

The dataset used in the experiments was released by the Wireless Sensor Data Mining (WISDM) Laboratory [32]. It includes raw data collected from several Android-based mobile devices under controlled laboratory conditions. In particular, the data refer to samples produced by the three-axis accelerometers embedded in smartphones, also capable to detect the device orientation. In the latest version of the dataset, thirty-six volunteers were engaged for collecting data. Each volunteer was asked to wear a smartphone in the front pocket of the pants while they performed specific actions for a given period of time. The horizontal movements of the user’s leg were captured by the X-axis of the accelerometer, whereas Y-axis captured the vertical movement, and finally, the forward and backward movements were captured by Z-axis. The labeling of the data, and the start and stop of the data collection related to any action were controlled by an app running on the smartphone, which also collected other information such as, timestamp, sample frequency, user’s name. Nevertheless, the app was set to collect data every 50 ms, that is $f=20 Hz$ which means 20 samples per second.

Six common activities were considered in data collection. They are:

21 $$\begin{aligned} A=\{Jogging,\;Walking,\;Upstairs,\;Downstairs,\;Sitting,\;Standing\} \end{aligned}$$

The dataset includes 1, 098, 208 samples, distributed as shown in Table 1. For any axis, the sample values range from $-20$ to 20.

**Table 1:** WISDM dataset distribution

Class	Samples	(%)
Jogging	342, 179	31.2
Walking	424, 399	38.6
Upstairs	122, 869	11.2
Downstairs	100, , 427	9.1
Sitting	59, 939	5.5
Standing	48, 395	4.4
Total	1, 098, 208	100

As it is usual, we performed an exploratory data analysis (EDA) [23], which led to eliminating some samples that report 0 values for all axes. Because the accelerometer is sensible to the gravity, the condition in which all axes release a value equal to zero is considered an error. The resulting distribution of the number of samples for any user is shown in Table 2.

**Table 2:** The WISDM dataset distribution for each user after EDA

User ID	Jogging	Walking	Upstairs	Downstairs	Sitting	Standing	Total
1	11,056	12,861	3120	2941	0	0	29,978
2	11,786	11,739	0	0	0	0	23525
3	11018	12,970	3411	3326	1609	2824	35,158
4	895	6079	1377	1761	1257	0	11369
5	6405	12,258	3387	3281	1664	1515	28,510
6	10961	12262	1661	1431	1679	709	28,703
7	9183	11,033	3601	2257	2529	2364	30,967
8	10,313	17108	4453	3345	2699	3269	41,187
9	0	12,923	0	0	0	0	12,923
10	12,084	13,046	4294	3792	0	1659	34,875
11	12,454	12,138	4392	2674	0	0	31,658
12	12,360	10,798	2654	2870	2289	1670	32,641
13	11,301	13,047	4637	4241	1179	1659	36,064
14	13,279	13,859	8179	2875	0	0	38192
15	10,800	11,468	2055	1762	0	0	26085
16	0	12,521	1411	1575	2984	1979	20,470
17	2887	9677	5689	3767	0	0	22,020
18	10,911	12,554	2409	2410	1467	1954	31,705
19	16,201	17,622	4280	2613	2534	2132	45,382
20	10729	13027	4833	4673	15,644	5389	54,295
21	9593	12,498	4841	4036	1609	2859	35,436
22	6224	7029	5430	3625	0	0	22,308
23	12,309	6589	4836	1939	0	0	25,673
24	12278	6256	3039	2929	690	544	25,736
25	6489	6979	0	0	0	0	13,468
26	11,,913	13,210	3618	3837	0	0	32,578
27	10,856	12,366	3064	3442	2099	1630	33,457
28	0	14,169	2892	2997	0	1300	21358
29	11,459	12,354	4769	4265	2319	1603	36769
30	0	12,579	4226	3872	1559	3099	25,335
31	14,075	16876	4679	3892	2148	2612	44,282
32	10,249	12,375	3797	2208	3059	1669	33,357
33	2946	14,898	2214	4535	3248	1612	29,453
34	12,869	13378	3921	2856	1575	1349	35,948
35	12,564	7162	0	0	1599	1069	22,394
36	11,887	6200	5429	4167	2500	1925	32,108
Total	330,334	423,908	122,598	100,194	59,939	48,394	1,085,367
%	30.4%	39.1%	11.3%	9.2%	5.5%	4.5%	100%

As depicted the total number of samples was reduced to 1, 085, 367, that is 12, 841 samples were deleted.

A dataset including transformed features is also provided by the WISDM Laboratory. It includes 46 features derived by considering a temporal window of 10 seconds, that is 200 sensor readings. These features are all derived from six basic features. For each axis they are: average value, standard deviation, average absolute difference, average resultant acceleration, time between peaks, and binned distribution. We remand to [32] for further details.

We remark that in this study, we do not use such transformed features, but we use the raw data. Nevertheless, as it will be shown in Sect. 5.5, we used such features for comparing the performance of our approach with the state-of-the-art based on Machine Learning techniques.

### HAR-images

The samples reported in Table 2 were used to build the dataset of HAR-Images as discussed earlier. In order to compare our approach with the state-of-the-art, we used a temporal window equal to $\varDelta t=10 \; seconds$, which corresponds to 200 samples ($\nu =200$) according to equation (2). Besides, a stride of 10 samples was used. As above discussed, because WISDM dataset includes data captured by a single three-axis accelerometer, we have $p=1$ and $q=3$. As a consequence, according to equation (7), for any $\varDelta t$ a single HAR-Image is produced ($p=1$) including 3 channels ($nC=3$). Besides, according to equation (20), the total number of 3-channels HAR-Images is equal to 105205, distributed as reported in Table 3. Finally, a quantization step of $Q=0.025$ was used, which led to having images of size equal to $(40 \times 40)$. Also, in order to adapt the entry values of the image-matrices to the DCNN input, all images were normalized to have entry values ranging from 0 to 1.

**Table 3:** The distribution of HAR-Images for any action

Class	HAR-Images
Jogging	32,439
Walking	41,719
Upstairs	11,666
Downstairs	9428
Sitting	5562
Standing	4391
Total	105,205

### DCNN model and experimental setting

**Fig. 5:**
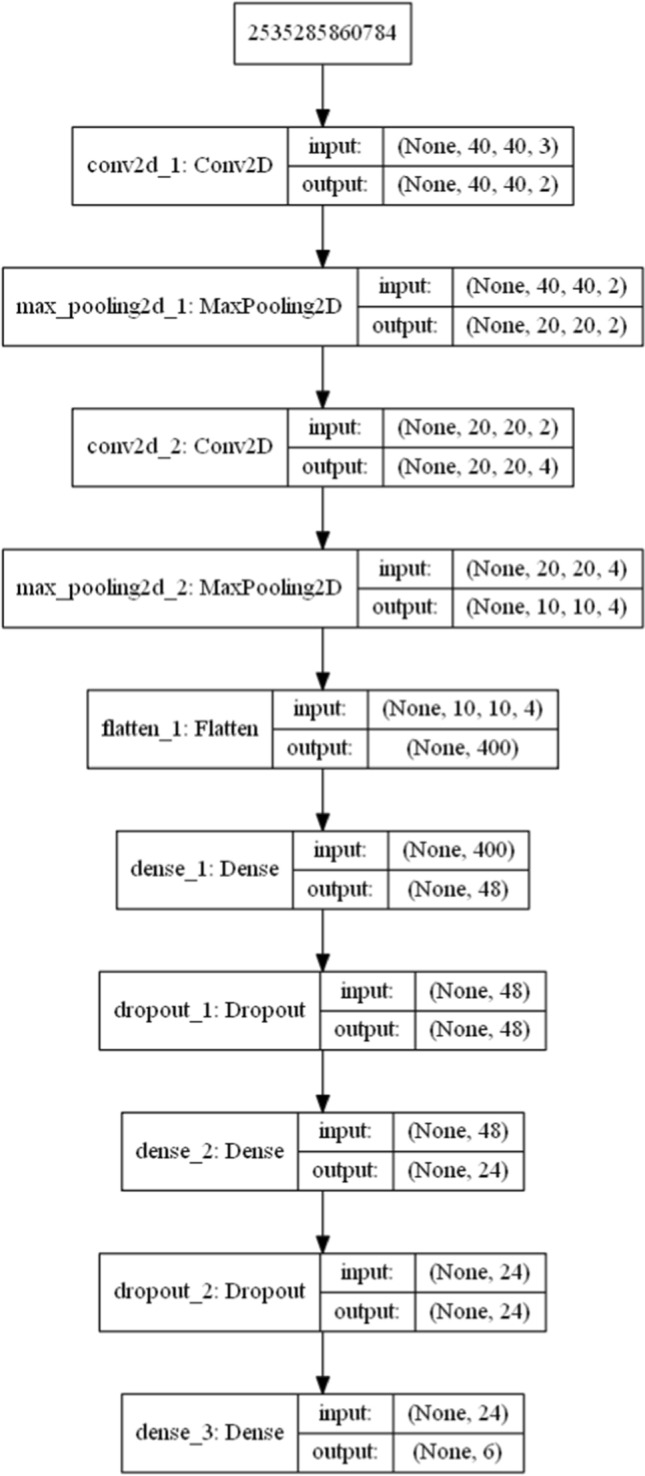
The Python DCNN structure

With reference to Fig. 4, the implemented DCNN structure includes two 3D-channels CNN-layers with 2 and 4 ($2 \times 2$)-dimensional filters, respectively, *padding=same*, *activation=relu*, and two ($2 \times 2$)-dimensional *MaxPooling* each for layer. Next, a flattening network is used, followed by a DNN. DNN includes two fully connected layers of 48 and 24 neurons, respectively. Both layers are followed by a Dropout of 0.5. Finally, a softmax function with 6 output concludes the network. Figure 5 shows the Python representation of the network structure along with the input and output sizes for each network element. The network was training by using *sparse-categorical-cross entropy* as loss function (equation (5)), *Adam* as optimizer, for 100 epochs with a $batch\_size=5$.

### Performance metrics and results

In order to evaluate the classification quality of the proposal, the following metrics were used on the testing dataset. They are derived by the multi-class confusion matrix [20]:

22 $$\begin{aligned} {\text {Accuracy}} \; {\text {(Acc)}}= & {} \frac{{\text {TP}}+{\text {TN}}}{{\text {TP}}+{\text {TN}}+{\text {FP}}+{\text {FN}}} \end{aligned}$$

23 $$\begin{aligned} {\text {Sensitivity}} \; {\text {(Sens)}}= & {} \frac{{\text {TP}}}{{\text {TP}}+{\text {FN}}} \end{aligned}$$

24 $$\begin{aligned} {\text {Specificity}} \; {\text {(Spec)}}= & {} \frac{{\text {TN}}}{{\text {TN}}+{\text {FP}}} \end{aligned}$$

25 $$\begin{aligned} {\text {Precision}} \; {\text {(Prec)}}= & {} \frac{{\text {TP}}}{{\text {TP}}+{\text {FP}}} \end{aligned}$$

26 $$\begin{aligned} {\text {AUC}}= & {} \frac{{\text {Sens}}+{\text {Spec}}}{2} \end{aligned}$$

27 $$\begin{aligned} F\_{\text {Measure}} \; {\text {(Fmea)}}= & {} \frac{2*{\text {Sens}}*{\text {Prec}}}{{\text {Sens}}+{\text {Prec}}} \end{aligned}$$

where for each class (action), TPs (True Positives) are the actions correctly classified, FPs (False Positives) are the actions incorrectly classified, FNs (False Negatives) are the actions incorrectly rejected, and TNs (True Negatives) are the actions correctly rejected. We remark that any metric ranges from 0 to 1 or equivalently from $0\%$ to $100\%$, where $100\%$ represents the best performance.

All the metrics were evaluated by using the Python library *scikit-learn* [19].

In order to investigate the performance of the proposal, without running into problems related to bias, the 10-fold cross-validation technique was used in the experiments. Accordingly, with reference to Table 2, the samples belonging to each action were first divided into 10 folds, and then, in turn, each fold was used for testing, while the remaining samples for training. To accomplish this, the *sklearn* library for Python was used.

Tables 4 and 5 show the confusion matrix and the metrics of the experiment results for any action, respectively. The, mean, standard deviation, and $95\%$ confidence intervals are also shown. As depicted, all the metrics achieved the maximum value for any action. Indeed, only a few samples were incorrectly classified (Table 4). Although some actions can be considered many similar, such as Sitting and Standing, the HAR-Images proved to be able to distinguish them with high accuracy. Indeed, the achieved accuracy was $100\%$. Also, the classifier showed some weakness for Upstairs, which, for a few cases, was confused with Walking or Downstairs. However, also in this case, the accuracy achieved values very close to $100\%$.

**Table 4:** Overall Confusion Matrix of the 10-fold cross-validation test

		Predicted					
		Jogging	Walking	Upstairs	Downstairs	Sitting	Standing
Actual	Jogging	32425	7	7	0	0	0
	Walking	0	41648	27	44	0	0
	Upstairs	37	73	11483	73	0	0
	Downstairs	5	58	65	9300	0	0
	Sitting	0	0	1	0	5542	19
	Standing	0	0	0	0	3	4388

**Table 5:** Metrics derived from the confusion matrix

	Acc	Sens	Spec	Prec	AUC	Fmea
Jogging	0.999	1.000	0.999	0.999	0.999	0.999
Walking	0.998	0.998	0.998	0.997	0.998	0.997
Upstairs	0.997	0.984	0.999	0.991	0.992	0.988
Downstairs	0.998	0.986	0.999	0.988	0.993	0.987
Sitting	1.000	0.996	1.000	0.999	0.998	0.998
Standing	1.000	0.999	1.000	0.996	1.000	0.997
Mean	0.999	0.994	0.999	0.995	0.997	0.994
st.d.	0.001	0.007	0.001	0.005	0.004	0.006
C.I. @ 95%	[0.997	[0.987	[0.998	[0.990	[0.993	[0.989
	1.000]	1.001]	1.000]	1.000]	1.000]	1.000]

### Comparison and discussion

In this Section, a comparison with some solutions presented in the literature is provided.

Firstly, in order to show the effectiveness of the HAR-Images as powerful features to be used in HAR, we tested the performance of the so-called *transformed features* used in the WISDM Lab [32]. We remark that, as aforementioned in Sect. 5.1, such transformed features are derived from the raw time-series data by using a temporal window of 10 seconds (corresponding to 200 samples), that is the same number of samples used in our experiments. The transformed dataset is provided from WISDM Lab as an ARFF file containing 46 (including the class) features and 5418 samples. Two features, the “UNIQUE_ID” and “user” were pruned in our experiments, because they could bias their results. Four classifiers from the WEKA suite [59] were used, that is Naive Bayes, MLP (Multi-Layer Perceptron), J-48 decision tree, and SMO (Sequential Minimal Optimization). Besides, the 10-fold cross-validation technique was also used to test the performance of the WISDM features.

**Fig. 6:**
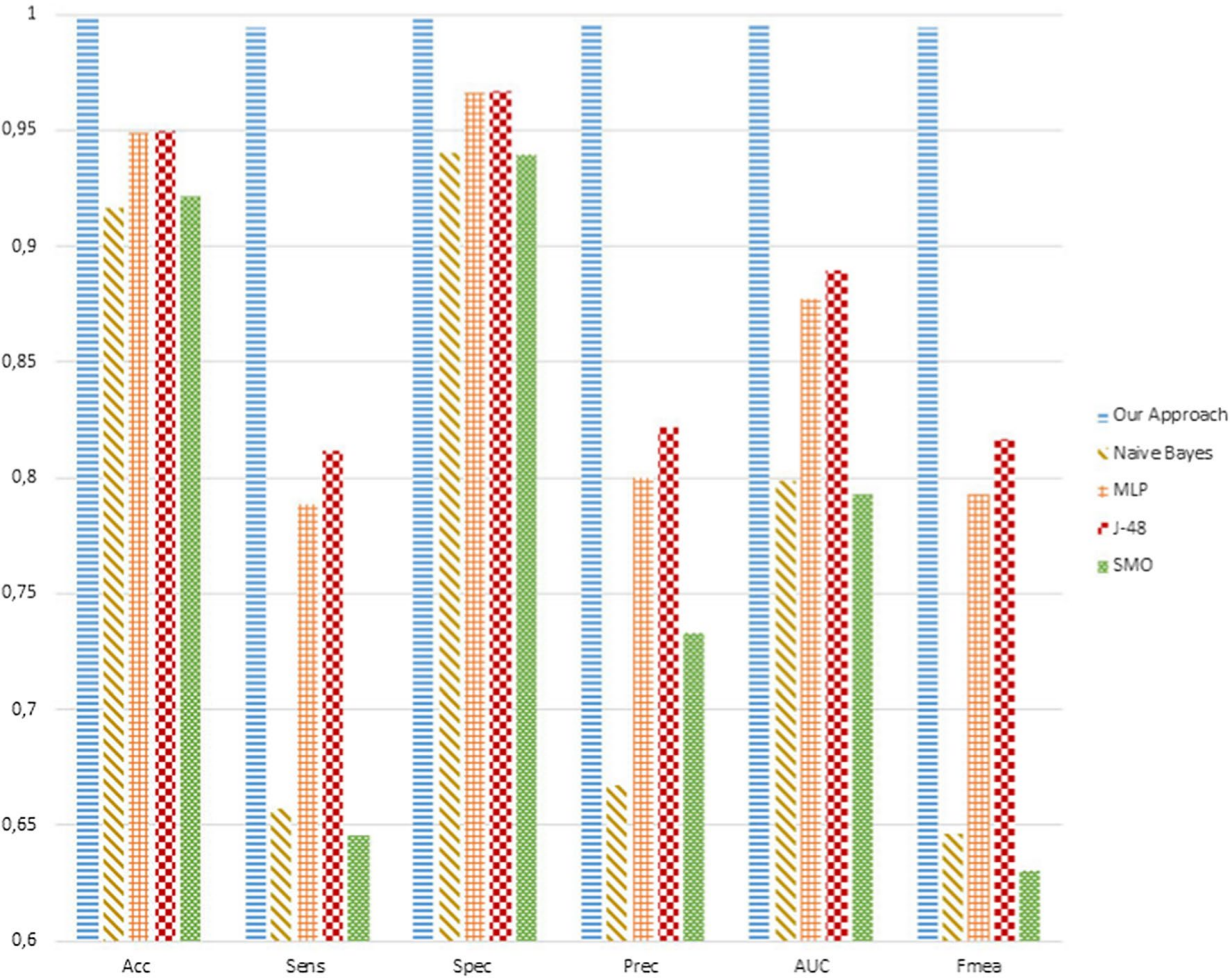
The 10-fold cross-validation results comparison on average. The WISDM transformed features are tested by using Naive Bayes, MLP, J-48, and SMO classifiers

Figure 6 depicts the average values of the performance metrics for any classifier. As shown, our approach outperformed all the others. All the metrics achieved a value very close to $100\%$. Although MLP and J-48 seem to perform better than Naive Bayes and SMO, their Sensitivity, of 0.78 and 0.81 respectively, is very low compared to the one achieved by our proposal, which is 0.99. Another important aspect that needs to be considered is that, unlike our approach, the transformed features seem to be unable to deal with unbalanced datasets. Indeed, as depicted in Table 6, the number of WISDM samples for any action is quite different. Most of the samples refer to the Walking and Jogging actions. The low values for Sensitivity, Precision, AUC, F-measure, and higher values for Specificity for the WEKA classifiers are a clear demonstration of this drawback introduced by the WISDM features.

Finally, in order to provide further proof of the effectiveness of our approach, Table 7 shows a comparison with some of the most notable solutions recently presented in the literature that use deep neural networks, that is DCNN [45], LSTM [46], and CNN [61]. DCNN makes use of merging features that include the WISDM transformed features with features extracted by a CNN. Notice that the accuracy of DCNN for any action is missing in the table and only the mean is reported, because we used the results published in their paper. LSTM refers to a solution based on a recurrent neural network, while CNN refers to a solution based on a pure CNN with a 1D convolution operation.

As depicted, our approach was able to recognize any action with a similar accuracy and precision. Indeed, all the metrics achieved similar value, almost $100\%$. On the contrary, Upstairs and Downstairs were confused by DCNN, LSTM, and CNN. Indeed, the associated metrics achieved the lowest values. Again, the Standing action was not well distinguished by other ones.

**Table 6:** The distribution of WISDM samples for any action

Class	Samples
Jogging	1625
Walking	2081
Upstairs	632
Downstairs	528
Sitting	306
Standing	246
Total	5418

**Table 7:** Average scores comparison of the 10-fold cross-validation test

		Jogg.	Walk.	Upst.	Downst.	Sitt.	Stand.	Mean
Acc	**Our Appr.**	0.999	0.998	0.997	0.998	1.000	1.000	**0.999**
	**DCNN** [45]	-	-	-	-	-	-	0.942
	**LSTM** [46]	0.987	0.977	0.958	0.965	0.994	0.994	0.979
	**CNN** [61]	0.985	0.963	0.952	0.956	0.991	0.991	0.973
Sens	**Our Appr.**	1.000	0.998	0.984	0.986	0.996	0.999	**0.994**
	**DCNN** [45]	0.939	0.977	0.723	0.923	0.801	0.921	0.881
	**LSTM** [46]	0.974	0.981	0.856	0.729	0.901	0.997	0.906
	**CNN** [61]	0.989	0.973	0.753	0.736	0.888	0.935	0.879
Prec	**Our Appr.**	0.999	0.997	0.991	0.988	0.999	0.996	**0.995**
	**DCNN** [45]	0.969	0.979	0.853	0.714	0.916	0.832	0.877
	**LSTM** [46]	0.985	0.961	0.787	0.868	0.994	0.885	0.913
	**CNN** [61]	0.961	0.931	0.856	0.814	0.961	0.845	0.895
Fme	**Our Appr.**	0.999	0.997	0.988	0.987	0.998	0.997	**0.994**
	**DCNN** [45]	0.953	0.978	0.776	0.800	0.834	0.857	0.866
	**LSTM** [46]	0.980	0.971	0.820	0.793	0.945	0.938	0.908
	**CNN** [61]	0.975	0.952	0.801	0.773	0.923	0.887	0.885

Bold refers to the maximum average value of each metric

## Conclusions and future works

In this study, we have proposed a method to enhance the performance of the COVID-19 tracking apps through the detection of human activity recognition (HAR). In particular, starting with raw data readings coming from built-in sensors in smartphones, we have derived special images, called HAR-Images, able to capture useful and salient knowledge related to the in-progress user activity, and thus to be used as a kind of signature of the activity. A deep convolutional neural network (DCNN) has been used to mine such insights. A mathematical model of the proposal has first been provided, and then its application on data coming from a three-axis accelerometer has been depicted.

Unlike the state-of-the-art approaches, which build images by using separately each axis of an accelerometer, the proposed HAR-Images are derived by exploiting contemporaneously a couple of axis. Their contents are used as coordinates of dots on an x-y plane. This different point of view confers to the proposal the capability to capture salient relations existing among data of the different axis gathered at the same time. As a consequence, the extracted images include more powerful insights, which allows building a more performant DCNN based classifier.

Indeed, the experimental results, obtained by using the WISDM dataset, have been absolutely satisfying. The metrics, derived by the multi-class confusion matrix of the 10-fold cross-validation test, have achieved very high values, $99.9\%$ on average. Compared with other state-of-the-art approaches, our proposal has proven to be the best. Indeed, the performance of most of the other approaches present in literature never have exceeded a F-measure of $91\%$.

These results have confirmed the effectiveness of the proposed HAR-Images and the DCNN structure in making HAR. In addition, its ability to working in real-time confers to it the possibility to be used for COVID-19 tracking apps.

The experimental results encourage us to investigate, in the future, the application of the HAR-Images also in other contexts, such as telemedicine or personal fitness monitoring.
